# Solitude and serotonin: juvenile isolation alters the covariation between social behavior and cFos expression by serotonergic neurons

**DOI:** 10.3389/fnins.2024.1446866

**Published:** 2024-10-22

**Authors:** Sarah E. D. Hutchens, Izza Khurram, Laura M. Hurley

**Affiliations:** ^1^Hurley Laboratory, Department of Biology, Indiana University, Bloomington, IN, United States; ^2^Center for the Integrative Study of Animal Behavior, Indiana University, Bloomington, IN, United States

**Keywords:** serotonin, dorsal raphe nucleus, social isolation, postweaning, social competence, social plasticity, opposite sex interaction, ultrasonic vocalization

## Abstract

Variation in the mutual responsiveness of social partners to each other can be reflected in behavioral suites that covary with neural activity in ways that track the salience or valence of interactions. Juvenile social isolation alters social behavior and neural activity during social interaction, but whether and how it alters the covariation between behavior and neural activity has not been as well explored. To address this issue, four classes of experimental subjects: isolated males, socially housed males, isolated females, and socially housed females, were paired with an opposite-sex social partner that had been socially housed. Social behaviors and c-Fos expression in the serotonergic dorsal raphe nucleus (DRN) were then measured in subjects following the social interactions. Relative to social housing, postweaning isolation led to a decrease in the density of neurons double-labeled for tryptophan hydroxylase and c-Fos in the dorsomedial subdivision of the DRN, regardless of sex. Vocal and non-vocal behaviors were also affected by isolation. In interactions with isolated males, both ultrasonic vocalization (USVs) and broadband vocalizations (squeaks) increased in conjunction with greater male investigation of females. Neural and behavioral measures also correlated with each other. In the isolated male group, the density of double-labeled neurons in the dorsomedial DRN was negatively correlated with USV production and positively correlated with a principal component of non-vocal behavior corresponding to greater defensive kicking by females and less investigation and mounting behavior. This correlation was reversed in direction for socially housed males, and for isolated males versus isolated females. These findings confirm that the dynamics of social interactions are reflected in c-Fos activation in the dorsomedial DRN, and suggest an altered responsiveness of serotonergic neurons to social interaction following social isolation in males, in parallel with an altered male response to female cues.

## Introduction

Social interaction is important to normal development. Social isolation in children and adolescents through circumstances like pandemic lockdowns or serious childhood illness are associated with physiological traits like increased cortisol levels (Taylor et al., 2022; de Laia Almeida et al., 2021) and mental health issues such as anxiety, depression, and reduced cognitive development (Manning et al., 2014; de Laia Almeida et al., 2021; Shah et al., 2020; Meherali et al., 2021; Pahl et al., 2021). These effects may endure past the time of the isolation itself (Loades et al., 2020). In animal models, social isolation in early life also has profound effects on adult social competence, in that previously isolated animals may be less responsive to the cues of social partners or show behaviors that are not adjusted to the varying dynamics of social interactions (Tóth et al., 2008; Oliveira, 2009; Taborsky and Oliveira, 2012; Keesom et al., 2017a; Maruska et al., 2019). Social isolation may increase aggressive behaviors, decrease or increase sociability, or alter the range of behaviors displayed in a given social context (Shimozuru et al., 2008; Bicks et al., 2020; Yamamuro et al., 2020; Araki et al., 2021). Sensitivity to isolation is also seen in communication behaviors. For example, social isolation may increase or decrease the numbers of vocal signals in social settings, change the relative production of different types or structural characteristics of vocalizations, or cause the production of vocalizations in abnormal contexts (Tomazini et al., 2006; Yu et al., 2011; Chabout et al., 2012; Hamed and Kursa, 2020; Caruso et al., 2022). Isolation changes not only the production of communication signals, but also responses to social signals by previously isolated individuals. Isolation may alter the attractiveness of vocal signals (Seffer et al., 2015), cause more rapid adaptation to aversive vocal signals (Hood et al., 2023), or lengthen the time needed to learn to distinguish among call types with different structure (Screven and Dent, 2019).

Isolation-induced changes in communication behavior may be brought about in part through effects of isolation on modulatory neurochemical systems (e.g., Hamed and Kursa, 2020; Broadfoot et al., 2023; Menon and Neumann, 2023). One of these is the serotonergic system, including the dorsal raphe nucleus (DRN). The DRN is a major source of serotonergic input to forebrain and midbrain regions, including sensory systems and regions related to social behavior (Waterhouse et al., 1986; Vertes, 1991; Hale and Lowry, 2011; Muzerelle et al., 2016; Niederkofler et al., 2016; Ren et al., 2018; Walsh et al., 2018; Steinbusch et al., 2021). Some DRN neurons are responsive to social context, and DRN neurons in turn influence social behaviors including aggression, sociability, and sexual behavior (Lowry et al., 2005; Liu et al., 2014; Niederkofler et al., 2016; Walsh et al., 2018; Petersen et al., 2020; Troconis et al., 2023). Manipulation of serotonin levels also alters the production of communication signals, and may selectively enhance behavioral responses to communication signals (Marquez and Chacron, 2020; Hood and Hurley, 2024). Social isolation affects many aspects of the serotonergic system, including the activity of DRN neurons during social interaction, the density of axons in target brain regions, the dynamics of serotonin release, and even the expression of serotonin receptors by postsynaptic neurons (Segal et al., 1973; Fulford and Marsden, 1998; Muchimapura et al., 2002; Schiller et al., 2003; Schiller et al., 2006; Bibancos et al., 2007; Ago et al., 2013; Dankoski et al., 2014). Although it is clear that social isolation alters both the serotonergic system and social behavior, whether and how isolation changes the relationship between serotonergic activity and social/communicative behaviors has been less well-explored.

To address these questions, we compared the effects of postweaning isolation on social behaviors, including vocal behaviors, and the activation of DRN neurons in male and female mice (*Mus musculus*) paired with a member of the opposite sex. In this context, males calibrate their vocal and non-vocal behaviors in response to female signals. When placed with female social partners, male mice produce the majority of ultrasonic vocalizations (USVs), which are attractive to females (Whitney et al., 1973; Hammerschmidt et al., 2009; Asaba et al., 2014; Neunuebel et al., 2015; Sterling et al., 2023). Females are the main producers of squeaks, also called broadband vocalizations (BBVs: White et al., 1998; Wang et al., 2008; Finton et al., 2017). Females produce BBVs in conjunction with kicking at males in the early stages of interactions (Finton et al., 2017). High numbers of early BBVs predict lowered male mounting later on, and the playback of BBVs to males temporarily suppresses USV production (Finton et al., 2017; Hood et al., 2023). Activity of the serotonergic system covaries with these behavioral events in both males and females. In the auditory midbrain of male mice, serotonin levels rise in the presence of a female partner, and inversely correlate with the number of female BBVs (Keesom and Hurley, 2016). Conversely, in subregions of the DRN that project to the auditory midbrain, numbers of neurons that are active during a social interaction in female mice correlate with the behavior of male social partners (Petersen et al., 2020). This system therefore provides an excellent platform for assessing the effects of social isolation on the correspondence between the activity of serotonergic neurons and social behavior.

Following several weeks of individual or same-sex group housing that began after weaning, male and female mice were placed with opposite-sex group-housed social partners, and the vocal and non-vocal behaviors displayed during this interaction were compared to the activation of serotonergic neurons in the DRN as measured through co-expression of the immediate early gene product c-Fos and tryptophan hydroxylase (TPH). We found that social isolation decreased the densities of serotonergic neurons expressing c-Fos in both sexes. Isolation also affected vocal and non-vocal behaviors by increasing both USVs and BBVs in parallel with greater male investigation of females. Isolation further reversed the correlation between c-Fos expression by serotonergic neurons and a principal component of behavior corresponding to female kicking, decreased anogenital investigation, and decreased mounting. These findings suggest an altered responsiveness of serotonergic neurons to social interaction following social isolation in males, in parallel with an altered male response to female cues.

## Methods

### Animals and housing

All experiments were conducted in accordance with the Guidelines for the Use of Laboratory Animals, and were approved by the Bloomington Animal Care and Use Committee (protocol 21-020). All mice were housed in standard mouse laboratory cages in the same room. Food and water were provided *ad libitum* with a light cycle of 14:10 h light:dark. Twenty male and twenty female CBA/J mice (the Jackson Laboratory, Bar Harbor, ME) were used as subjects, and an additional 6 males and 6 females were pair- housed and used only as non-subject social partners in behavioral trials. Mice were shipped from Jackson labs at 3 weeks as non-littermates. No fighting was observed when mice were co-housed.

### Experimental overview

**Figure 1:**
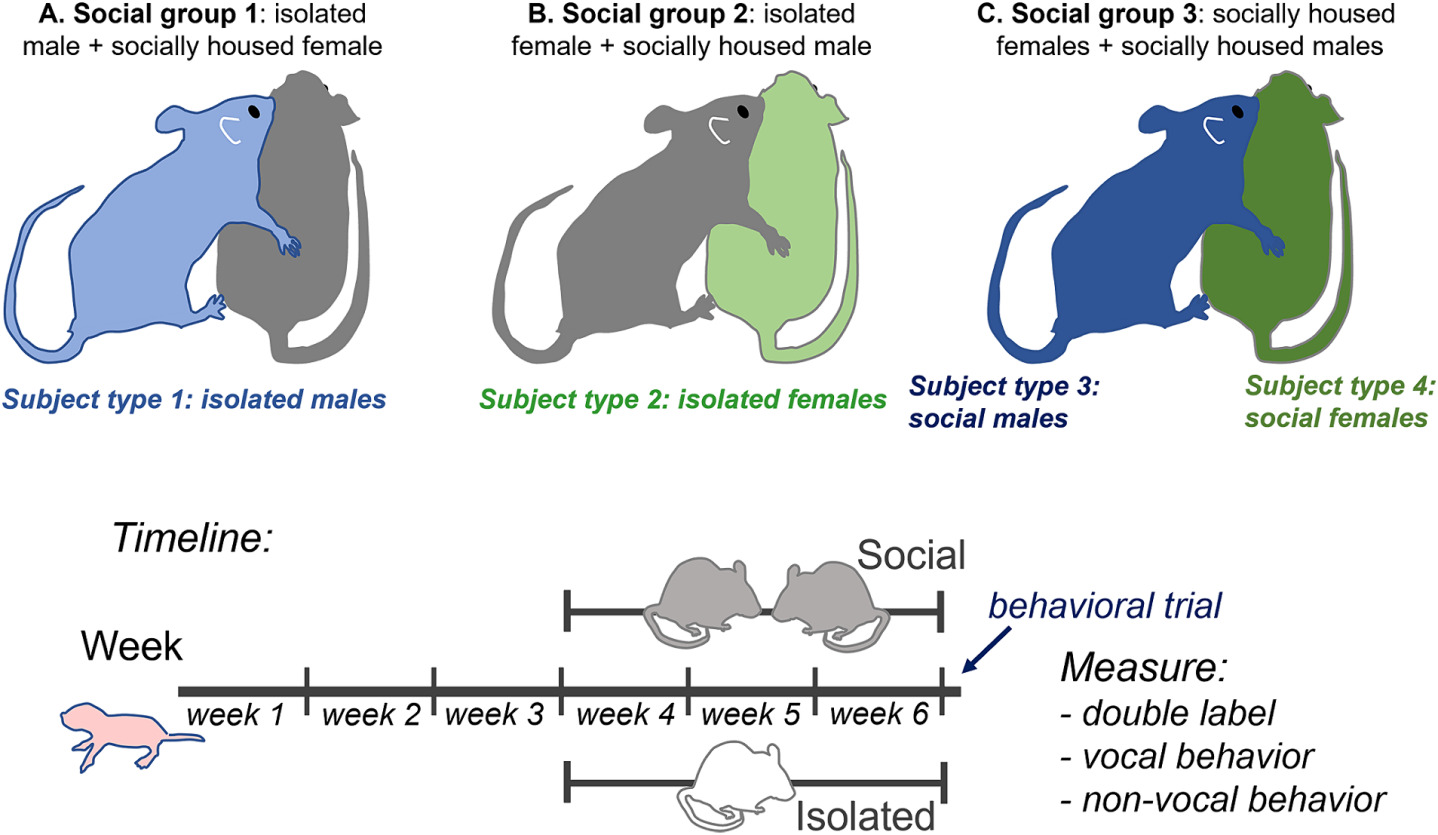
*Top*: Diagram of experimental design showing the three social treatment groups **(A–C)** that result in four subject groups: 1. isolated males (light blue), 2. isolated females (light green), 3. socially housed males (dark blue), and 4. socially housed females (dark green), each interacting with a socially housed partner. Some behavioral measurements that could not be ascribed to a single individual, such as call number and nose-to-nose investigation, are the same for social males and females in the same specific interaction. *Bottom*: timeline of experimental manipulations.

The 20 male and 20 female mice arrived at 3 weeks of age and were placed in either individual or same-sex paired housing (*n* = 10 each for each sex). Pair-housed mice were placed with cagemates of approximately similar weight. Mice stayed in their social treatment group for 21–25 days prior to behavioral trials. This resulted in four *subject groups* of individually housed (isolated) and socially housed (social) males and females. Following the housing treatment, mice were placed with a social partner of the opposite sex for a 60-min behavioral trial in which mice interacted freely. All social partners of the opposite sex were pair-housed with same-sex cage mates. This resulted in three *social treatment groups* with isolated male subjects (paired with group housed female non-subjects), isolated female subjects (paired with group housed male non-subjects), and socially housed male and female subjects paired with each other. These groups are depicted in Figure 1 (top). Following social interaction, subjects were perfused, and brains were sectioned and labeled with antibodies for tryptophan hydroxylase (TPH) and c-Fos. The numbers of double-labeled neurons in different subregions of the dorsal raphe nucleus (DRN) were compared to vocal and nonvocal behaviors across groups and individuals.

### Behavioral trials

Two to four days prior to behavioral trials, mice were habituated by twice being placed in their home cages for a period of 30 min in the sound attenuation chamber (IAC Acoustics, Naperville, IL) in which behavioral trials occurred. Behavioral trials took place between the hours of 9:30 a.m. and 3:00 p.m. On the day of behavioral trials, individual mice were habituated in a sound-attenuation chamber for a minimum of 40 min before trials. They were then transferred to another sound-attenuated behavioral recording chamber into a clean cage with pine bedding to allow for behaviors like digging. An opposite-sex social partner that had been housed in a same-sex pair was introduced into the cage. For all subject mice, this was the first postweaning encounter with a member of the opposite sex. Males in all groups directed behaviors toward females within seconds. Subjects were allowed to freely interact with social partners for a period of 1 h. Video recordings of non-vocal behaviors were made with a Canon VIXIA video camera above the cage (Q-See, Digital Peripheral Solutions Inc., Anaheim, CA) with a Q-See four-channel DVR PCI video capture card. The camera was attached to a Scepter Model E22 Monitor for viewing on the outside of the isolation chamber. Vocal behaviors were recorded using a condenser microphone (CM16/CMPA, Avisoft Bioacoustics, Glienicke/Nordbahn, Germany) placed above the cage. Audio recording was controlled through an Avisoft-UltrasoundGate 116H Recorded (#41163; Avisoft Bioacoustics, Berlin, Germany) with a sampling rate of 250 kHz. This was attached to a Dell Optiplex 960 Computer running Avisoft Recorder Software and a 16-bit condenser microphone (CM16, CMPA; Avisoft Bioacoustics, Berlin, Germany; 200 kHz maximum range). Following the one-hour interaction, mice were transferred to the holding chamber for 30 min to allow for optimal expression of c-Fos (Petersen et al., 2020), followed by perfusion with 4% paraformaldehyde in physiological saline. Brains were sectioned and immunohistochemically processed to label c-Fos, an immediate early gene product, and tryptophan hydroxylase (TPH), a synthetic enzyme for serotonin (see Figure 1, bottom, for experimental timeline).

### Dominance testing

Dominance testing in pair-housed subjects and sham dominance testing were performed 1–2 days before behavioral trials and on the morning of behavioral trials to ensure consistency. To ascertain dominant versus subordinate status, cage mates were placed in an arena containing a tube wide enough for only one mouse to travel through it. Bedding from the cage of opposite-sex mice was placed in the bottom of the tube. Mice were simultaneously released at either side of the tube. The trial ended when one mouse (the subordinate) left the tube, or after 5 min had elapsed. Sham dominance testing was accomplished by placing single housed mice in the same apparatus, with their own bedding in the tube. This allowed for similar handling to the socially housed mice.

### Measuring estrous phase

The estrous stages of female subjects and social stimulus females were assigned by characterizing cells collected by vaginal lavage on the day of the behavioral trial (Hanson and Hurley, 2012). Following lavage, collected cells were smeared onto a glass slide and labeled with Giemsa stain. Presence of cornified epithelial cells or of both cornified and nucleated epithelial cells indicated proestrus or estrus, while the presence of leukocytes indicated diestrus (Goldman et al., 2007). Females in proestrus or estrus were combined into one group since receptive sexual behavior occurs in both of these stages, and females in metestrus and diestrus were similarly combined (Rodgers, 1970; Fitzroy Hardy, 1972). Estrous staging was additionally performed in the 3 days prior to behavioral trials to promote accurate staging.

### Immunohistochemistry

**Figure 2:**
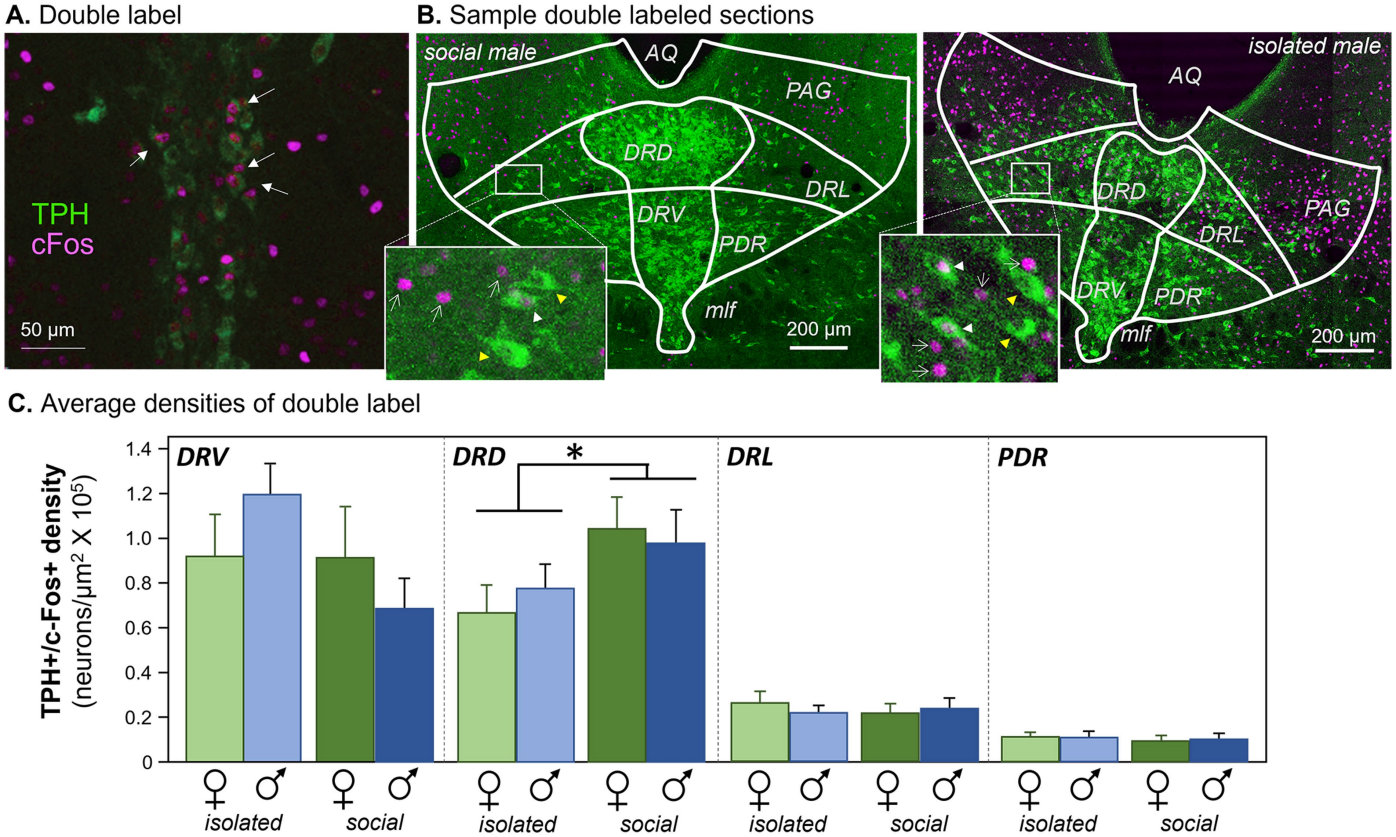
Antibody labeling. **(A)** Double label of antibodies to tryptophan hydroxylase (TPH) and cFos in the DRN. Arrows indicate neurons with both labels. **(B)** Sample images showing TPH label from each of the four subject groups. White arrowheads indicate double-labeled neurons. AQ, aqueduct; PAG, periaqueductal gray; DRD, dorsomedial dorsal raphe; DRV, dorsoventral dorsal raphe; DRL, dorsolateral dorsal raphe; PDR, posterodorsal raphe; MLF, median longitudinal fasciculus. Images have been enhanced in brightness and contrast for the figure. Insets: white arrowheads indicate double-labeled neurons, yellow arrowheads indicate neurons labeling for TPH but not c-Fos, and white arrows indicate c-Fos-labeled nuclei. **(C)** Densities of neurons labeled for antibodies to c-Fos and TPH in four subregions of the dorsal raphe nucleus. The four subject groups of individually versus socially housed females and males are represented for each subregion. *p < 0.05.

Following the one-hour interaction, mice were transferred to the holding chamber for 30 min to allow for optimal expression of c-Fos (Petersen et al., 2020), followed by perfusion with 4% paraformaldehyde in physiological saline. Following perfusion in 4% paraformaldehyde solution, brains were postfixed overnight, then placed in 30% sucrose in phosphate-buffered saline (PBS) until equilibrated. Brains were embedded in Tissue Tek (Sakura Finetek USA, Torrance, CA) and cut at 50 μm thickness on a sliding freezing microtome (American Optical Company, Buffalo, NY). Sections were collected throughout the rostral-caudal extent of the DRN from ~ Bregma = 5.02 mm to ~ Bregma = 4.24 mm (Paxinos and Franklin, 2004). Sections were collected into cryoprotectant solution and stored in a freezer at −80°C until immunohistochemically labeled. Sections were thawed and rinsed in PBS for 5 × 5 min, then incubated for an hour in blocking solution of 10% donkey serum in PBS with 0.3% Triton X (PBSTx). After blocking, sections were incubated in antibodies to TPH (1:500 mouse monoclonal T0678, lot 069M4768V: Millipore-Sigma, Burlington, MA) and c-Fos (1,1,000 rabbit polyclonal 226,003, lot 9-89: Synaptic Systems, Goettingen, Germany) diluted in PBS with 5% donkey serum and 0.3% Triton X overnight on an orbital shaker at ~4°C. Sections were then rinsed in PBS with 5% donkey serum for 4 × 5 min and incubated with fluorescently labeled secondary antibodies (1,333 Alexa Fluor 488 donkey anti-rabbit, A21206, lot 2,229,195 and 1,200 Alexa Fluor 680 donkey anti-mouse, A10043, lot 2,165,747, Thermo-Fisher Scientific, Waltham, MA) in PBS with 5% donkey serum and 0.3% Triton X for 2 h, after which they were rinsed 3 × 10 min in PBS. Following the final rinse, sections were mounted in glass slides, coverslipped with ProLong Gold anti-fade mounting medium (Thermo-Fisher Scientific, Waltham, MA), and imaged. This process resulted in nuclei labeled for c-Fos and cell bodies labeled for TPH (Figure 2A). Controls for the c-Fos label consisted of omitting primary and pre-adsorbing the primary antibody by incubating it with antigenic peptide. c-Fos-labeled nuclei were not observable in control treatments.

### Image collection

Sections were imaged at 10X on a Leica SP8 scanning confocal microscope in the Light Microscopy Imaging Center (LMIC) at Indiana University. Alexa Fluor 488 and 680 fluorophores were simultaneously excited using 488 and 680 nm laser lines. Twelve z planes separated by 2.41 um were collected through each section.

### Image analysis

Image stacks were pre-processed in FIJI to split the imaging channels and create merged projection images of the 12 z-planes. Cell nuclei positive for c-Fos (c-Fos+) were marked in ImageJ using a macro-based neurohistological analysis suite developed by Timothy and Forlano (2019) that identified c-Fos + nuclei in regions of interest based on a thresholding function. Regions of interest consisted of subregions of the DRN as described in Petersen et al. (2020). Briefly, boundaries for DRN subregions at specific rostrocaudal levels were derived from plates 73, 71, 68, and 66 the mouse brain atlas (Paxinos and Franklin, 2004). Boundary guides were fitted to match the chemoarchitecture of TPH-positive (TPH+) neurons relative to landmarks including the cerebral aqueduct and the median longitudinal fasciculi. Boundary diagrams were fitted to each section in Photoshop and superimposed on images in ImageJ to define regions of interest. DRN subregions for which ROIs were defined consisted of two densely clustered midline regions and two more dispersed lateral regions. The midline subregions were the dorsally located DRD (dorsal subnucleus of the dorsal raphe) and the ventrally located DRV (ventral subnucleus of the dorsal raphe). The lateral subregions were the DRL (dorsolateral subregion of the dorsal raphe), and the PDR (posterior-dorsal raphe subnucleus, located adjacent to the DRV).

After c-Fos + nuclei were identified and marked, images containing the locations of marked c-Fos + nuclei were imported to Photoshop for comparison with TPH+ cell bodies. Neurons were considered to be double-labeled if c-Fos + nuclei and darker regions within TPH+ neurons indicative of nuclei coincided. This estimation of double label was conducted manually because the high density and overlap of TPH+ neurons in midline subnuclei of the DRN make automation of this function difficult. Counts of double-labeled neurons across all sections within a specific subregion for a given subject were then normalized to the combined areas of the corresponding regions of interest to achieve an estimate of density. The numbers of TPH+ neurons were estimated by counting nuclei without label surrounded by cell bodies immunopositive for TPH. An average of 3.2 ± 0.12 sections per individual were analyzed. These ranged from 1 to 4 (1 section: *n* = 1; 2 sections: *n* = 5; 3 sections: *n* = 19; 4 sections: *n* = 15). Cell counts and areas from different sections were summed for specific DRN sections to obtain a single measure of density per individual per DRN subregion.

### Behavioral measurement

Vocal and nonvocal behaviors were scored by researchers blind to the experimental groups of the subjects. Nonvocal behaviors were scored from video recordings of each interaction using Behavioral Observation Research Interactive Software (BORIS v. 7.13.6; Friard and Gamba, 2016). Behaviors were sampled in every other five-minute period, so that a total of 30 min of the one-hour interaction were scored. Technical issues prevented one video recording from saving in the isolated female group. Tails of mice were marked using nontoxic ink to clearly distinguish males from females during analysis. Five behaviors were scored in both sexes. Investigation was divided into *nose-to-nose investigation*, consisting of mutual facial exploration rostral to the eyes, and *anogenital investigation*, in which one mouse investigated the hindquarters of the other near the tail or under a rear leg. *Grooming* was defined as any time there was a back-and-forth motion of the mouse’s head on its fur, clear paw movement over its ears, pulling of tail or leg up, reaching around, and/or scratching. *Rearing* included any time the mouse was on its hind legs touching the wall with at least a 45-degree angle with one or two paws on the side of the cage. *Digging* was defined as any instance when the alternation of paws of the mouse caused the movement of bedding while there was no locomotion. Two behaviors were sex-specific. *Mounting* was defined as one individual grasping the other with its forelegs, often accompanied by pelvic thrusting. Only males showed this behavior. In contrast, only females *kicked* at males, usually following anogenital investigation by males. Kicks were often paired with BBVs, sudden darts and lunging after or before kicking, and were often performed from a curled posture. Durations and numbers of events were scored for digging, grooming, both types of investigation, and rearing, while the highly transient events of kicking and mounting were only scored as the number of events.

Vocal behavior was analyzed using Raven sound analysis software (Raven Pro 1.6). Spectrograms of the audio recordings were created and the male ultrasonic vocalizations (USVs) and female BBVs were counted. USVs were further separated into two categories. Nonharmonic USVs had single observable harmonics around 70 kHz, while harmonic USVs had prominent harmonics at higher and lower frequencies. Harmonic vocalizations are enriched around mounting and are sensitive to past social isolation (Hanson and Hurley, 2012; Keesom et al., 2017a).

### Statistical analysis

Statistics were performed with SPSS 29.0 (IBM, Armonk, NY). Densities of neurons double labeled for c-Fos and TPH were assessed across subregions of the DRN using a repeated measures General Linear Model with subregion as a within-subjects variable and social treatment and sex as between-subjects factors. There were no effects of dominance status on either densities of double labeled neurons (one-way ANOVAs, *p* > 0.5) or vocal behaviors (independent samples Kruskal-Wallis tests, *p* > 0.2 for all for either males or females). There were no effects of estrous phase of females or of the female partners of males on either densities of double labeled neurons (one-way ANOVAs, *p* > 0.2) or vocal behaviors (independent samples Kruskal-Wallis tests, *p* > 0.3 for all calls). Because of the lack of effect of dominance status and estrous phase on these measurements, these factors were not included in the final models.

Numbers of vocalizations were log transformed after adding one to each value. Even after this transformation, numbers of both types of USVs and BBVs were not normally distributed in all social treatment groups (Shapiro–Wilk tests, *p* < 0.05). Kruskal-Wallis tests were therefore performed to assess differences in the calls across treatment groups. To compare vocal behavior to the densities of double-labeled neurons, an analysis of covariance (ANCOVA) was performed on the ratios of harmonic to nonharmonic call number versus double-labeled neuron densities for the DRD, across the four subject groups. Using the ratios compensated for different baseline calling rates among males, and normalized the distribution of values. For the two social subject groups (male and female), vocalization values were the same, since these values came from the same interactions for male and female social pairs. Spearman’s correlations were used to assess correlations between harmonic and non-harmonic vocalization categories, and between vocalizations and non-vocal behaviors.

Because of non-normality in some nonvocal behaviors even when log transformed, independent Kruskal- Wallis tests were used to compare the total durations of nonvocal behaviors across the three social groups, and the Benjamini-Hochberg correction was used to adjust for multiple comparisons. Behaviors tested in this way were female and male digging, grooming, anogenital investigation, rearing, nose-to-nose investigation (the same values for both sexes), and male mounting and female kicking.

In order to compare nonvocal behaviors to the densities of double-labeled neurons in the DRN, we first reduced the dimensionality of the multiple nonvocal behaviors. Because of non-normal distributions of kicking and mounting, we used a categorical principal components analysis (CATPCA). Both male and female behaviors across all social treatment groups were incorporated into the CATPCA, since male and female behaviors may influence each other. This analysis was based on the numbers rather than durations of behaviors, to make measurements of kicking and mounting more comparable to other behaviors. Log transformed (log +1) counts for all behaviors except kicking and mounting were normally distributed (Shapiro-Wilks tests, *p* > 0.05), and these were coded as numerical variables. Numbers of kicks and mounts were not normally distributed (Shapiro-Wilks tests, *p* < 0.05), and were coded as categorical variables after adding one to each value. The object scores from dimension 1 and dimension 2 of the CATPCA were then compared to the densities of double labeled neurons within each of the four social treatment groups using Pearson’s correlations.

## Results

### Social treatment, but not sex, affects c-Fos expression by serotonergic neurons

The densities of serotonergic neurons expressing the immediate early gene product c-Fos following an opposite-sex interaction were assessed across four subject groups that differed in sex and social history. These groups were socially housed females (social females), females that were individually housed after weaning (isolated females), socially housed males (social males), and individually housed males (isolated males). During behavioral interactions, each of these types of subjects was placed with a socially housed partner of the opposite sex. Socially housed male and female subjects were paired with each other during the opposite-sex interaction. This created a total of three types of social treatment groups: isolated male subjects (with socially housed female partners that were not used for immunohistochemical studies), isolated female subjects (with socially housed male partners that were not used for immunohistochemical studies), and socially housed male and female subjects that were both used for immunohistochemical studies (Figure 1, top).

Densities of serotonergic neurons that expressed c-Fos following the social interaction, as seen in Figure 2A, were measured in four subregions of the dorsal raphe nucleus (Figure 2B): the midline dorsal subdivision (DRD), the midline ventral subdivision (DRV), the dorsolateral subdivision (DRL), and the posterodorsal subdivision (PDR). Across the four dorsal raphe subregions, there were significant differences in the densities of double-labeled neurons among the subregions themselves (repeated measures General Linear Model with subregion as a within-subjects variable and social treatment and sex as between-subjects factors: *F*(_3, 32_) = 49.881, *p* < 0.001). The two medial dorsal raphe regions had higher densities than the two more lateral subregions (Figure 2C). There was also an interaction between the subregion and social treatment (*F*(_3, 33_) = 5.970, *p* = 0.002), but no interactions between subregion and sex or among subregion, social treatment, and sex (*p* > 0.05). Within individual subregions, only the DRD showed a significant effect of social treatment on density (Least Significant Difference *post hoc* tests, *p* = 0.042 for the DRD, *p* > 0.05 for the DRV, DRL, and PDR). This analysis shows that social isolation affected the densities of serotonergic neurons expressing c-Fos, and this effect was only observed in the DRD subdivision, regardless of sex.

A similar trend was observed when the percentages of double-labeled neurons relative to the total numbers of TPH-labeled neurons were used rather than neuron density. There was a significant effect of subregion, with the midline divisions having a higher percentage of double-labeled neurons (repeated measures General Linear Model with subregion as a within-subjects variable and social treatment and sex as between-subjects factors: *F*(_3, 33_) = 30.161, *p* < 0.001). There was also an interaction between the subregion and social treatment (*F*_(3, 33)_ = 5.411, *p* = 0.004), but no interactions between subregion and sex or among subregion, social treatment, and sex (*p* > 0.1). Although no individual DRN subregion showed a significant effect of social treatment, there was a strong trend in the DRD (Least Significant Difference *post hoc* test, *p* = 0.055), but not for other regions (*p* > 0.2 for the DRL, DRV, and PDR). Counts of all c-Fos + neurons regardless of serotonergic status differed among subregions (repeated measures General Linear Model with subregion as a within-subjects variable and social treatment and sex as between-subjects factors: *F*_(3, 33)_ = 28.707, *p* < 0.001), but there were no effects of social treatment, sex, or the interaction of these factors (*p* > 0.1 for these comparisons). Overall, the general patterns of differences between socially housed and isolated individuals were similar for measurements of both the density and relative percentage of serotonergic neurons expressing c-Fos.

These differences due to social treatment were not attributable to differences in the numbers of TPH+ neurons. Numbers of TPH+ neurons did show an effect of DRN subregion (repeated measures General Linear Model with subregion as a within-subjects variable and social treatment and sex as between-subjects factors: *F*_(3, 33)_ = 99.952, *p* < 0.001), and also an interaction between subregion and social treatment (*F*_(3, 33)_ = 2.963, *p* = 0.046). However, the only subregion showing an effect of social treatment on the number of TPH+ neurons was the DRV (Least Significant Difference *post hoc* test, *p* = 0.003), not the DRD or other subregions (*p* > 0.1). The number of TPH+ neurons also showed an interaction between subregion, sex, and social treatment (*F*_(3, 33)_ = 4.09, *p* = 0.014). This arose from significantly lower numbers of TPH+ neurons for females relative to males in the DRD and DRV subregions, but only in the isolated social treatment groups (Supplementary Figure S1A, Least Significant Difference *post hoc* test, *p* = 0.017 for isolated females versus males in the DRD and *p* = 0.042 for isolated females versus males in the DRV). There were no sex differences in any other subregions or social treatment groups (*p* > 0.2 for all other comparisons). This sex difference was likely due to an increased number of TPH+ neurons seen in isolated males but not females, only in the DRD and DRV subregions (Supplementary Figure S1B, Least Significant Difference *post hoc* test, *p* = 0.039 for isolated versus social males in the DRD and *p* < 0.001 for isolated versus social males in the DRV). Thus, although numbers of TPH+ neurons were affected by social treatment and sex, these differences had no direct parallels in the effects of social treatment on the densities or percentages of double labeled neurons across social groups and subregions.

Because serotonergic and non-serotonergic neurons are interconnected in the DRN (e.g., Rood and Beck, 2014), we also compared the ratios of double-labeled neurons in different subregions to the total numbers of c-Fos-positive nuclei. In this analysis, there were also differences among DRN subregions (repeated measures General Linear Model with subregion as a within-subjects variable and social treatment and sex as between-subjects factors: *F*_(3, 33)_ = 135.047, *p* < 0.001), and an interaction between the subregion and social treatment (*F*_(3, 33)_ = 5.662, *p* = 0.003). However, in *post hoc* analysis, the only significant difference between socially housed and isolated groups was for the DRV subregion (Least Significant Difference *post hoc* test, *p* = 0.006), but not for other regions (*p* > 0.1 for the DRD, DRL, and PDR). In contrast to other measures of c-Fos activity, the values for socially housed mice were lower than for isolated mice (Supplementary Figure S2). This suggests that relatively fewer serotonergic neurons than non-serotonergic neurons in the DRV express c-Fos following social interaction in socially housed mice compared to isolated mice.

There were no effects of male dominance status (one-way ANOVA, *n* = 5 dominant and 5 subordinate individuals, *F*_(1, 9)_ = 10.41, *p* = 0.532) or female dominance status (one-way ANOVA, *n* = 5 dominant and 5 subordinate individuals, *F*_(1, 9)_ = 4.34, *p* = 0.665) on double-labeled neuron density in the DRD. There were also no effects of the estrous phase of females (one-way ANOVA, *n* = 10 in diestrus/metestrus and 9 in estrus/proestrus, *F*_(1, 18)_ = 0.02, *p* = 0.89) or of the estrous phase of female partners of males (one-way ANOVA, *n* = 11 in diestrus/metestrus and 8 in estrus/proestrus, *F*_(1, 18)_ = 1.76, *p* = 0.203).

### Social treatment affects vocal behavior

**Figure 3:**
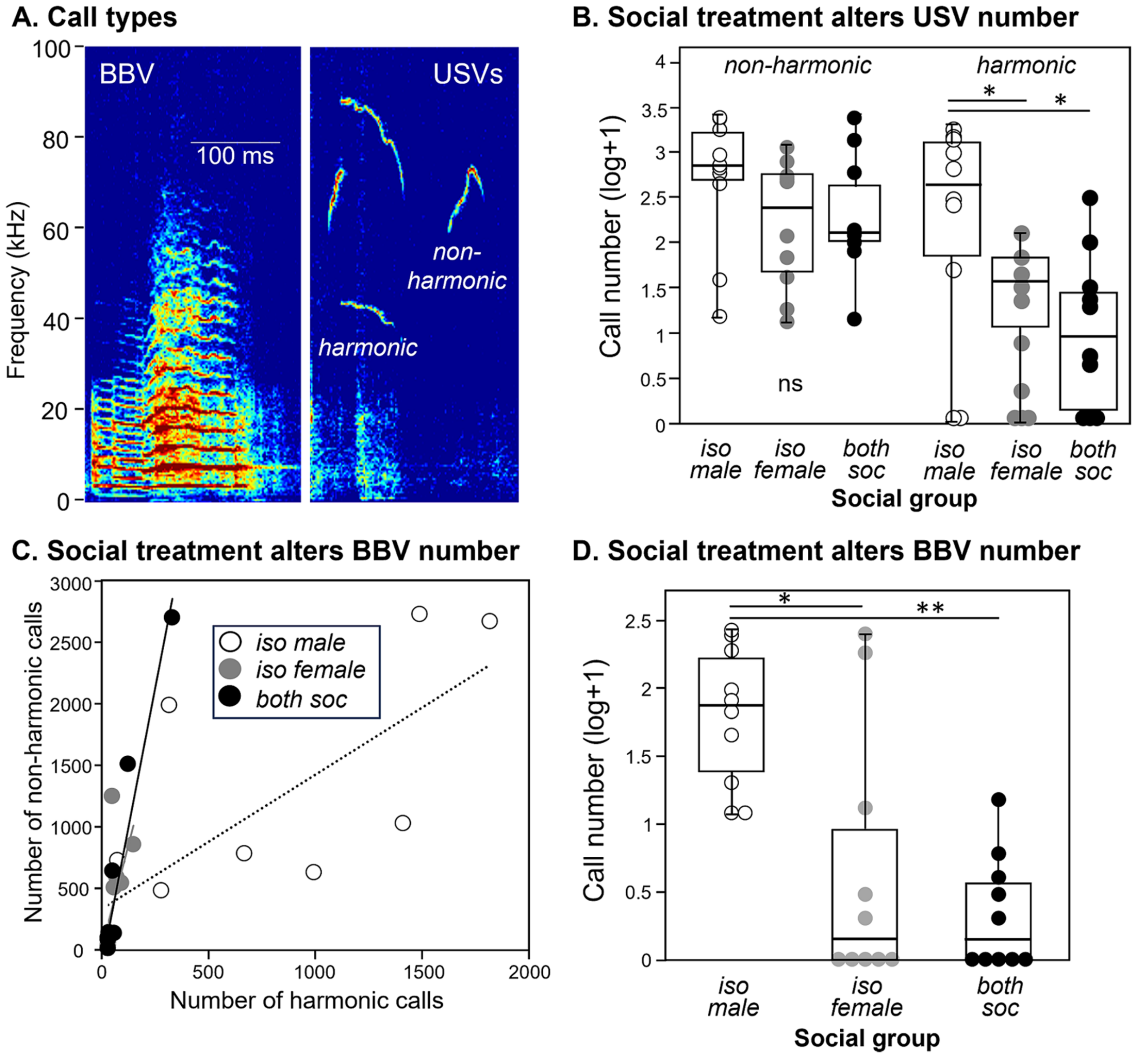
Differences in calls across the three different social groups. **(A)** Spectrograms showing examples of a BBV (left) and harmonic and non- harmonic USVs (right). **(B)** The numbers of harmonic USVs, but not non-harmonic USVs, are higher in groups with individually housed males. Values are shown as log transformations to better compare call types. **(C)** Numbers of harmonic versus nonharmonic USVs are correlated in the three social groups. Harmonic and nonharmonic calls are significantly correlated in all groups. **(D)** The numbers of BBVs are higher in groups with individually housed males. Identical values are slightly displaced from each other to distinguish all measurements. *p < 0.05; **p < 0.001.

Two classes of vocal behaviors were recorded during opposite-sex social interactions. Ultrasonic vocalizations (USVs) are produced predominantly by males during opposite-sex interaction, although females produce a minority of these calls (Neunuebel et al., 2015; Sterling et al., 2023). We divided USVs into two additional categories: “harmonic” USVs, and “non-harmonic” USVs. As the name suggests, harmonic USVs show prominent harmonics at around 40 and 60–80 kHz, while non-harmonic USVs show a single harmonic at around 70 kHz (Figure 3A, right). Harmonic USVs are enriched in the times around male mounting behavior (Hanson and Hurley, 2012). We also measured broadband vocalizations (BBVs), which are human-audible calls with pronounced harmonic structure, also called low frequency harmonic calls (LFHs: Grimsley et al., 2013) or squeaks (Figure 3A, left). BBVs are produced by females in several contexts, including when females direct kicks and lunges at males, and when females are being mounted by males (White et al., 1998; Wang et al., 2008; Sugimoto et al., 2011; Finton et al., 2017). The production of calls was measured across the three social treatment groups.

Call production depended on social treatment and call type. The number of non-harmonic USVs did not vary among treatment groups (Figure 3B, left; independent samples Kruskal-Wallis test, *H* (2) = 3.089, *p* = 0.213). In contrast, harmonic USVs varied significantly across the three social treatment groups (Figure 3B, right; independent samples Kruskal-Wallis test, *H* (2) = 7.102, *p* = 0.029), with the isolated male social group producing higher numbers of harmonic calls than the isolated female group (*p* = 0.021) or the social group (*p* = 0.021). This demonstrates that the social housing treatment specifically influenced the production of harmonic but not non-harmonic USVs, with the isolated male social group highest in the number of harmonic USVs. Numbers of harmonic and nonharmonic USVs correlated with each other within each group (Spearman correlations, isolated male group: *r_s_* = 0.839, *p* = 0.002, isolated female group: *r_s_* = 0.853, *p* = 0.002, social group: *r_s_* = 0.926, *p* < 0.001), although the proportion of harmonics was higher in the male isolated group than the other groups (Figure 3C). The number of female-produced BBVs were also different across the three social treatment groups (Kruskal-Wallis test, *H* (2) = 13.223, *p* = 0.001), with a larger number of BBVs in the isolated male social group than in the isolated female group (*p* = 0.015) or the social group (*p* = 0.002; Figure 3D).

The numbers of total USVs were not affected by male dominance status (independent samples Kruskal-Wallis test, *n* = 10 dominant and 10 subordinate individuals, *H* (2) = 1.46, *p* = 0.23) or by female dominance status (independent samples Kruskal-Wallis test, *n* = 10 dominant and 10 subordinate individuals, *H* (2) = 0.21, *p* = 0.65). Total USV numbers were also not affected by the estrous phases of females (independent samples Kruskal-Wallis test, n = 10 in diestrus/metestrus and 9 in estrus/proestrus, *H* (2) = 0.027, *p* = 0.87) or by the estrous phases of female partners of males (independent samples Kruskal-Wallis test, *n* = 11 in diestrus/metestrus and 8 in estrus/proestrus, *H* (2) = 0.825, *p* = 0.364).

### Vocal production correlates with DRD density for isolated but not socially housed males

**Figure 4:**
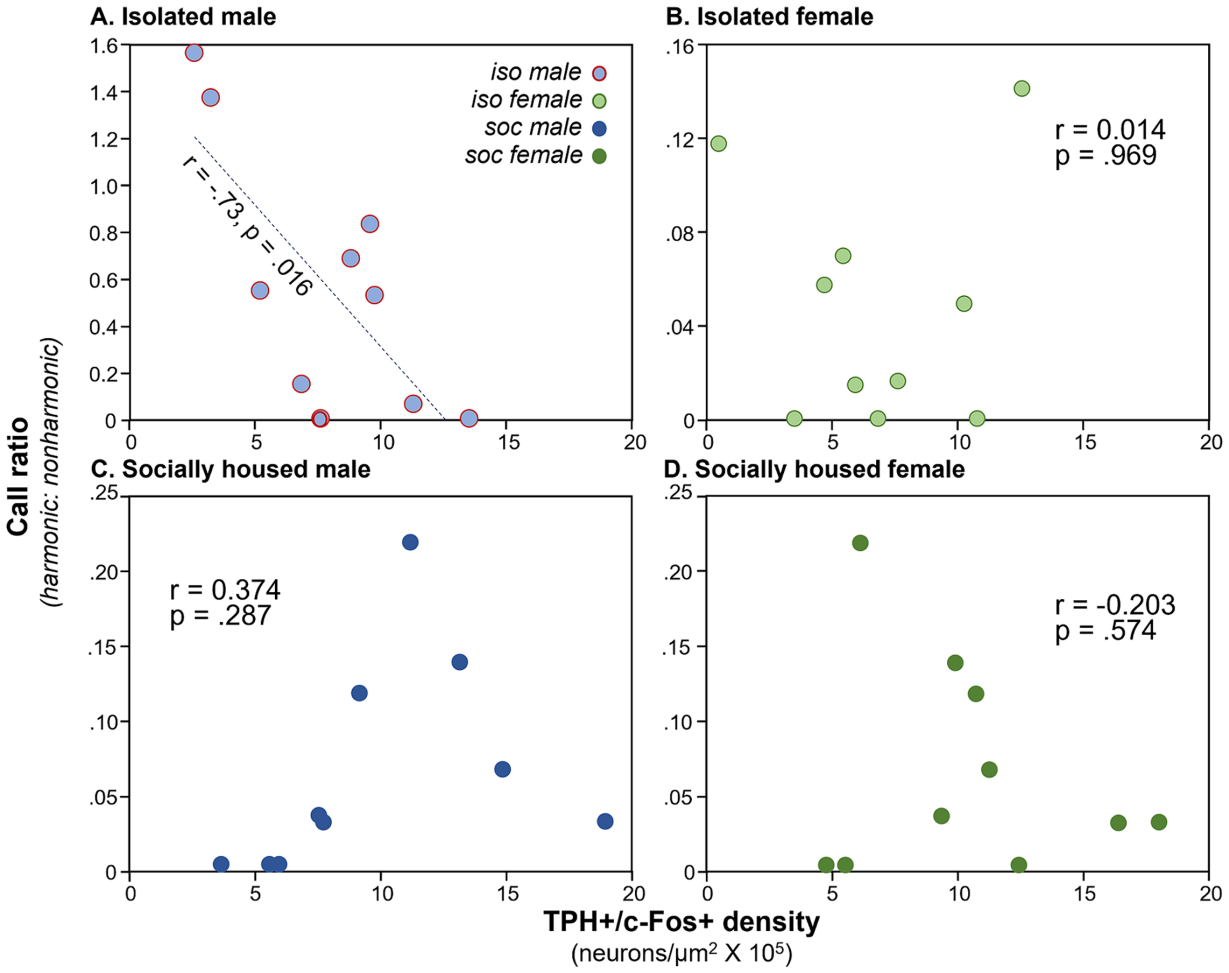
Plots of call ratio (numbers of harmonic/non-harmonic calls) for the four subject groups versus numbers of neurons expressing the double label, across individual mice. **(A)** Isolated males, **(B)** isolated females, **(C)** social males, and **(D)** social females (dark green). Note the difference in Y-axis values across groups. Y-axis values for groups **(C,D)** are the same for individual males and females that were in the same social interaction.

We have previously observed correlations between communication behavior and serotonergic activity (Keesom and Hurley, 2016), so we compared densities of double-labeled neurons in the DRN with the production of USVs. We used the ratio of harmonic to non-harmonic calls as a metric to account for variation in overall calling rate among males in order to normalize for different overall calling rates among individuals. A significant correlation only occurred for the DRD subregion. There was a main effect of subject type on harmonic ratio (ANCOVA, *F*_(3, 32)_ = 18.82, *p* < 0.001), with the highest harmonic ratio seen for isolated males. There was also a significant correlation of the harmonic ratio with the density of double-labeled neurons (*F*_(1, 32)_ = 10.88, *p* = 0.002). Finally, there was an interaction between DRD density and subject type (F_(3, 32)_ = 9.72, *p* < 0.001), suggesting different relationships between DRD density and call ratio among groups. To follow up on this, Pearson’s correlations were performed for each subject group, showing that the isolated male group showed an inverse correlation between DRD density and call ratio (Figure 4A: *r* = −0.734, *p* = 0.016), but the other types of subjects did not (Figures 4B–D: isolated females: *r* = 0.014, *p* = 0.969; social males: *r* = 0.374, *p* = 0.287; isolated females: *r* = −0.203, *p* = 0.574). The three subregions of the DRN other than the DRD did not show significant covariation between harmonic ratio and neuron density, or interactions between neuron density and subject groups, although the PDR and DRV showed differences in harmonic ratio among subject groups due to isolated males producing proportionally more harmonic calls (Table 1).

**Table 1:** Results of ANCOVAs comparing harmonic ratios across subject groups with the densities of double-labeled neurons as a covariate for different DRN (dorsal raphe nucleus) subregions.

DRN region	DRD		PDR		DRL		DRV	
	*F*	*p*	*F*	*p*	*F*	*p*	*F*	*p*
Subject group	18.819	<0.001	4.039	0.015	2.521	0.076	8.878	<0.001
Density	10.877	0.002	0.082	0.777	0.401	0.531	4.404	0.044
Group*density	9.717	<0.001	0.228	0.876	0.258	0.855	3.57	0.025

Red values were significant after correction for multiple comparisons. DRD = dorsomedial subregion; PDR = posterodorsal region, lateral to the midline; DRL = lateral subregion; DRV = ventromedial subdivision.

### Non-vocal behaviors change with social treatment

**Figure 5:**
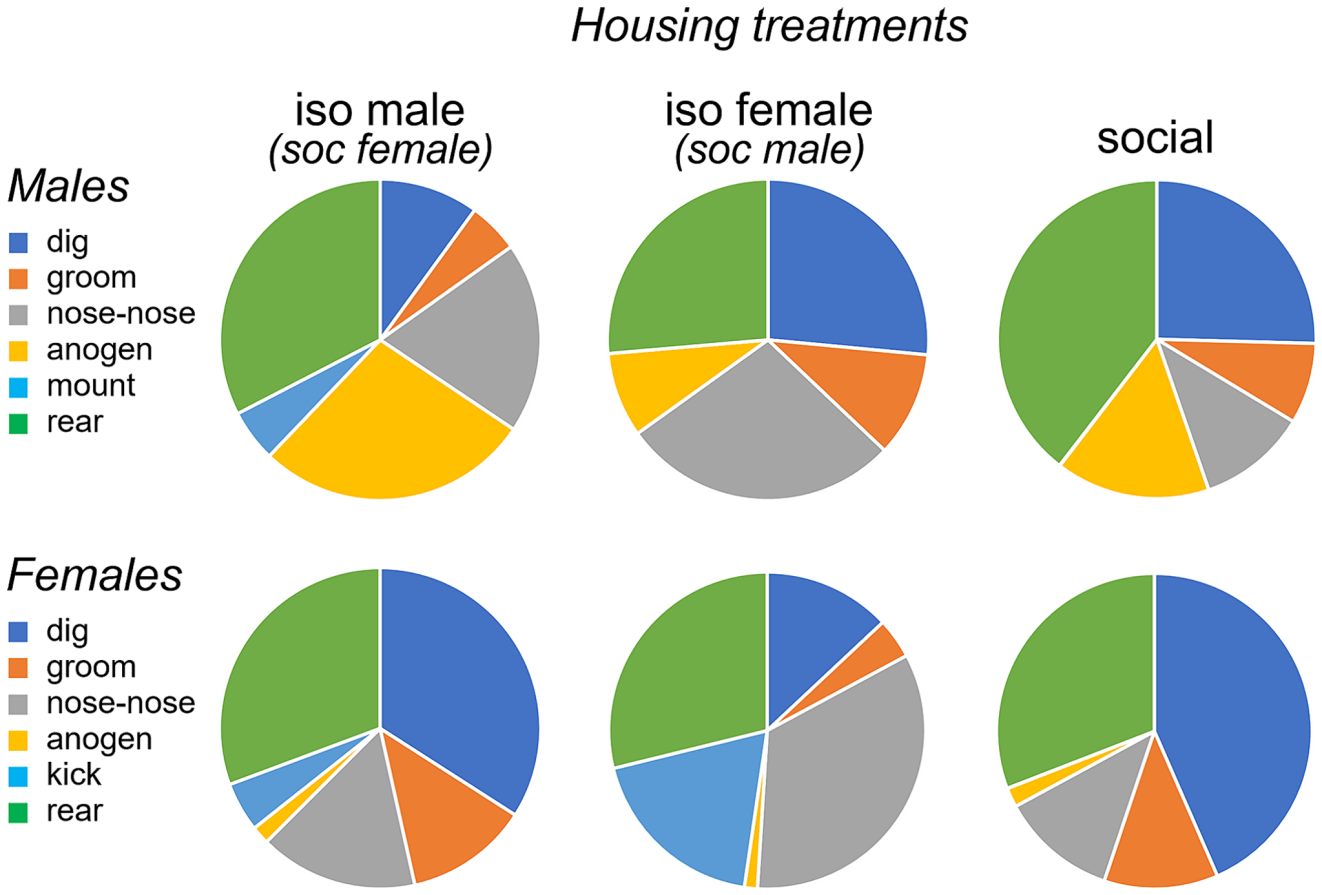
Pie charts showing proportions of time spent performing different behaviors by males (top row) and females (bottom row) during an opposite-sex social interaction. The three social treatment conditions of isolated males (left), isolated females (middle) and socially housed males and females (right) are shown. Females in the isolated male treatment and males in the isolated female treatment were socially housed. “nose-nose” = nose-to-nose investigation; “anogen” = anogenital investigation.

In addition to vocal behaviors, we measured non-vocal behaviors: the duration of digging, grooming and rearing, and the social behaviors of nose-to-nose investigation and anogenital investigation. We also measured the numbers of two sex-biased behaviors: mounting by males and kicking by females. Suites of male and female behaviors varied across the three social treatment groups. Qualitatively, the isolated male/social female group was characterized by relatively high levels of anogenital investigation by males, mounting by males, and kicking by females (Figure 5, left column). Even higher levels of female kicking occurred in the isolated female/social male group, although no mounting and relatively little male anogenital investigation were observed (Figure 5, middle column). In the all-social group, little kicking or mounting occurred (Figure 5, right column). Non-vocal behaviors correlated with vocal behaviors (Spearman’s correlations, Supplementary Table S1). Both classes of USV correlated with mounting and anogenital investigation by males, and BBVs correlated positively with mounting and kicks by females, and negatively with digging by males. These correlations support previous findings of vocal signals during opposite-interaction being related to sexual behavior, or responses to sexual behavior.

**Figure 6:**
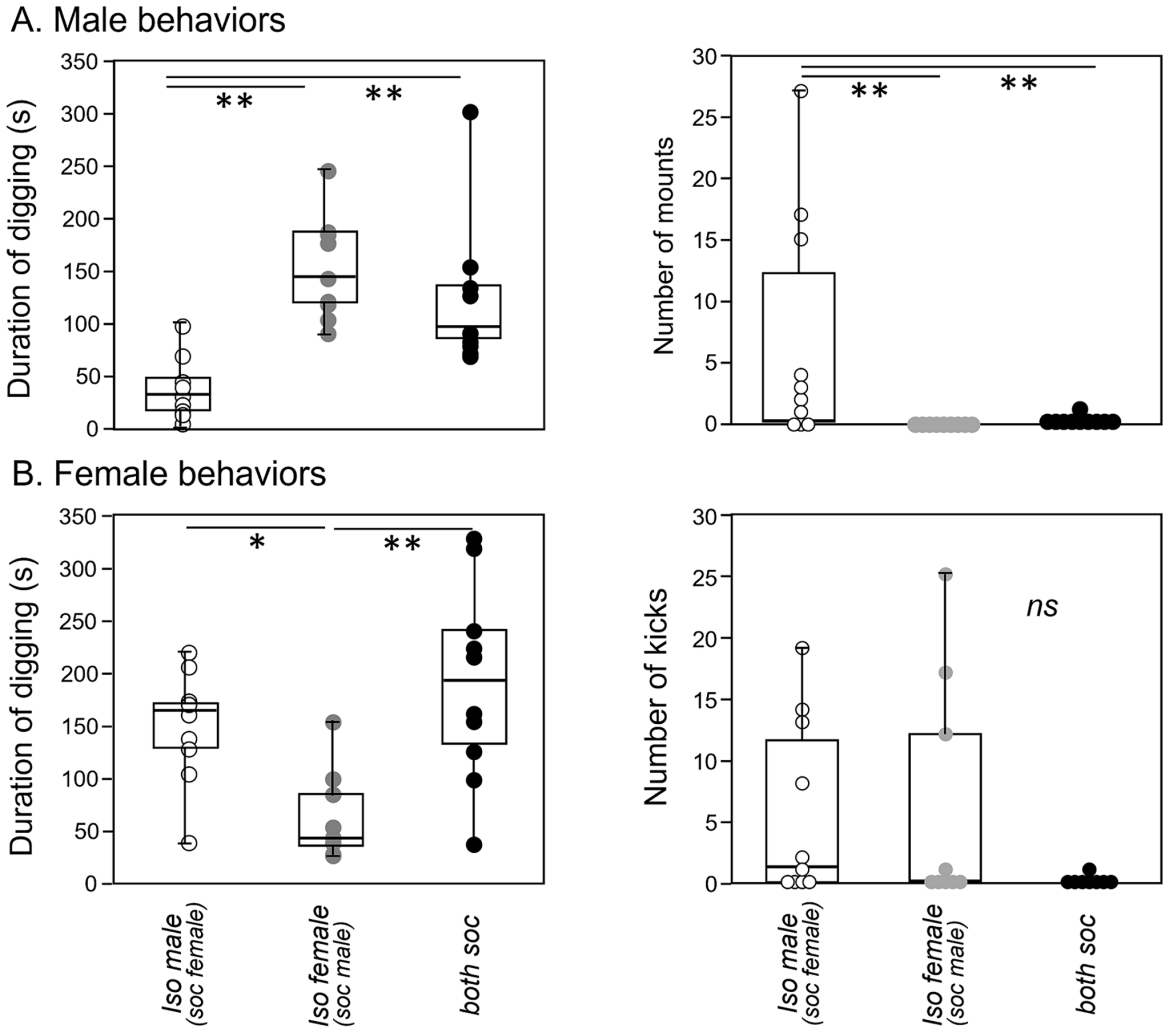
Box-and-whiskers plots of behaviors shown by males and females during an opposite-sex social interaction. **(A)** Isolated males show a lower total duration of digging (left panel) and a higher number of mounts (right panel) than the socially housed males in the other two treatment groups. **(B)** Isolated females also show a lower total duration of digging (left panel) than the socially housed females in the other treatment groups. Although numbers of kicks by females are higher in both of the isolated groups than the social group (right panel), this difference is not significant. *p value of less than 0.05, **p value of less than 0.01.

Quantitatively, there was significant variation among social treatment groups for some behaviors in males (Table 2). Among the measured behaviors, digging and mounting were different among social treatment groups (independent samples Kruskal-Wallis test; digging: *H* (2) = 17.871, *p* < 0.001; mounting: *H* (2) = 14.256, *p* < 0.001). Isolated males dug less than socially housed males in the other two groups (Figure 6A, left; *p* < 0.001 versus isolated female group, *p* = 0.005 for social group). In contrast, isolated males mounted more than the other two groups (Figure 6A, right; *p* < 0.001 versus isolated female group, *p* = 0.002 versus social group). Male anogenital investigation was not significantly different among groups after correction for multiple comparisons.

**Table 2:** Results of Kruskal-Wallis tests for behavioral differences among social interaction groups.

Male behaviors	*H*	*p*	Female behaviors	*H*	*p*
*dig*	17.87	<0.001	*dig*	12.27	0.002
*groom*	2.1	0.35	*groom*	3.58	0.167
*nose-nose*	5.12	0.08			
*anogen*	8.02	0.018	*anogen*	0.09	0.96
*rear*	5.14	0.08	*rear*	0.21	0.9
*mount*	14.26	<0.001	*kick*	5.77	0.06

Nose-nose values are identical for males and females since it is a joint behavior. “Anogen” = anogenital investigation of the social partner.

For females, the only behavior that differed significantly among groups was digging (Figure 6B, left, Table 2, independent samples Kruskal-Wallis test; digging: *H* (2) = 12.273, *p* = 0.002). Similar to males, the group with an isolated female showed reduced digging relative to the other two groups (*p* = 0.007 versus isolated male group, *p* < 0.001 versus social group). Although kicking occurred in a higher number of females in the isolated male-social female and isolated female- social male groups, this difference was not significant, although it showed a strong trend (Figure 6B, right; Kruskal-Wallis test, *H* (2) = 5.765, *p* = 0.056).

### Non-vocal behaviors correlate with DRD activity

**Figure 7:**
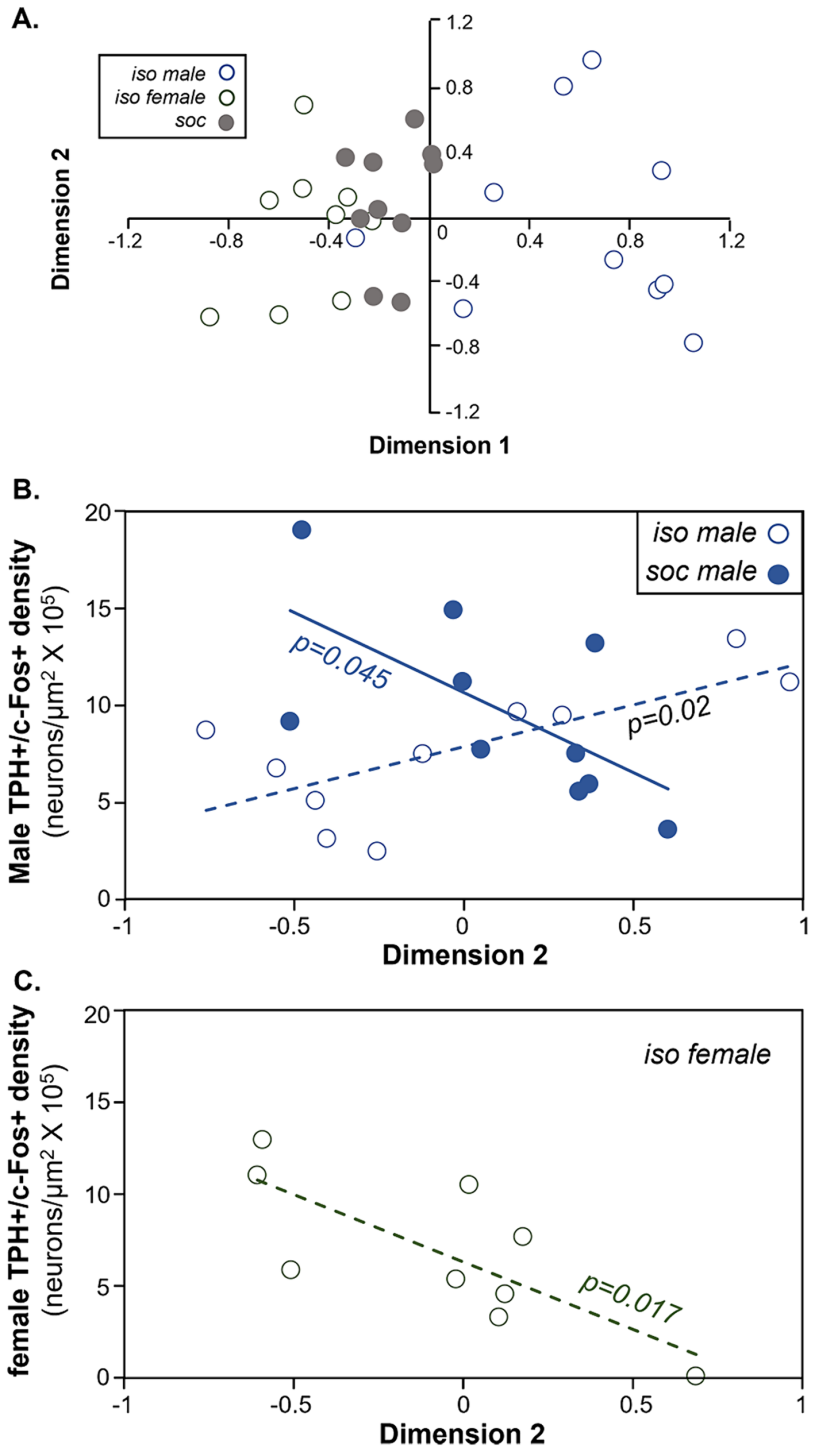
Comparison of the density of double labeled neurons in the DRD and behavior during the social interaction. **(A)** Plot of the first versus second dimensions of a categorical PCA of males and female behaviors. Open blue circles = the isolated male group. Open gray circles = the isolated female group. Filled gray circles = the social group. **(B)** Comparison of the densities of double-labeled neurons in the DRD versus dimension 2 scores of the categorical PCA in males. Values correlate positively across isolated males (open circles) but negatively across socially housed males (closed circles). **(C)** Comparison of the densities of double-labeled neurons in the DRD versus dimension 2 scores of the categorical PCA in females. Values are negatively correlated in isolated females.

To assess whether non-social behaviors correspond to c-Fos activity of serotonergic neurons, we reduced the dimensionality of all non-vocal behaviors measured for both males and females with a principal components analysis. To more closely compare all behaviors including kicking and mounting, the numbers of all behaviors were used as variables. Because kicking and mounting were not normally distributed even when transformed, we performed a categorical principal components analysis, in which some variables were continuous and some were categorical. To do this, the behaviors of digging, grooming, rearing, and anogenital investigation were log transformed, creating normal distributions, and were coded as continuous variables. Numbers of kicks and mounts were coded as categorical/ordinal variables. The first two dimensions of the extraction accounted for 47.83% of the variation in behaviors. A scatterplot of these first two dimensions of the analysis shows the isolated male group (open circles blue outline) and the isolated female group (open circles gray outline) as the most distinct, with the social treatment group (gray circles) in the middle (Figure 7A). Table 3 shows how different behaviors loaded onto Dimension 1 and Dimension 2. Factors that loaded strongly onto Dimension 1 were male digging (“mdig” negatively loading), nose-to-nose investigation (“inn”), anogenital investigation of females by males (“miag”), mounting (“mount”), and female digging (“fdig”). Dimension 1 thus corresponded to male sexual interest. Factors that loaded strongly onto dimension 2 were male rearing (“mrear”), female anogenital investigation (“fiag”, negatively loading), and female kicking (“kick”).

**Table 3:** Component loadings for the CATPCA.

Behaviors	Dim 1	Dim 2
*mdig*	−1.664	−0.04
*mgroom*	−0.535	−0.848
*inn*	1.052	0.934
*miag*	1.402	−0.365
*mrear*	−0.61	1.569
*mount*	1.496	−1.01
*fdig*	1.138	0.233
*fgroom*	0.966	0.902
*fiag*	−0.175	−1.536
*frear*	0.013	0.962
*kick*	0.045	1.281

“m” and “f” designations indicate sex of the animal performing the behavior. Mounts were exclusively male and kicks were exclusively female. “inn” = nose-nose inspection, “iag” – anogenital inspection.

For both groups of male subjects and for isolated females, the density of double-labeled neurons correlated with the object scores of Dimension 2 of the CATPCA (Pearson correlations, *p* > 0.05, Table 4). These relationships are also seen in Figures 7B,C. Figure 7B depicts the correlation between Dimension 2 and the densities of double-labeled neurons in the DRD. For social males (filled blue circles), the correlation between these variables was negative, so that higher values of Dimension 2 were correlated with lower densities of double-labeled neurons (Pearson correlation, *r* = −0.644, *p* = 0.045). For isolated males (open circles), the correlation was negative (Pearson correlation, *r* = 0.715, *p* = 0.02). For the isolated female group, Dimension 2 was also negatively correlated with the density of double-labeled neurons (Pearson correlation, *r* = −0.762, *p* = 0.017; Figure 7C). Thus, the direction of the correlations between Dimension 2 and the density of serotonergic neurons expressing c-Fos was different for socially housed versus isolated males, and for isolated males versus isolated females.

**Table 4:** Correlations between the object scores for the first two dimensions (Dim 1 and 2) of the principal components analysis and densities of serotonergic neurons expressing c-Fos.

	iso male		iso female		soc male		soc female	
	*r*	*p*	*r*	*p*	*r*	*p*	*r*	*p*
Dim 1	−0.168	0.643	−0.334	0.379	0.018	0.962	−0.067	0.854
Dim 2	0.715	0.02	−0.762*	0.017	−0.644*	0.045	−0.581	0.078

iso = isolated; soc. = socially housed.

## Discussion

Postweaning social isolation has well-documented effects on adult social behavior and on the function of social regulatory neural systems (Shimozuru et al., 2008; Bicks et al., 2020; Yamamuro et al., 2020; Araki et al., 2021), but how social isolation affects the relationship between these is not well understood. Predictions for the effects of social isolation on DRN-behavior covariation include a consistency in the underlying relationship that extends to a more extreme range representing altered behavioral norms, a diminished correlation reflecting a decreased effect of the social environment, or a correlation in a different direction that could correspond to an altered relevance of a given social situation. In the current study, postweaning isolation in mice altered c-Fos expression by serotonergic neurons in the DRD, but not other subdivisions of the DRN, in conjunction with social behaviors including vocal communication. Behaviors were correlated with c-Fos expression by serotonergic neurons, but social isolation reversed the direction of the correlation. Here, we discuss the nature of these relationships and their significance for the influence of juvenile social isolation on the regulation of social flexibility.

### Effects of social isolation across groups

The serotonergic system is highly sensitive to postweaning social isolation (Fone and Porkess, 2008; Lukkes, 2009; Keesom and Hurley, 2020), which has wide ranging effects on TPH activity, serotonergic axon density in target brain regions, serotonin release/reuptake, and serotonin receptor expression (Segal et al., 1973; Fulford and Marsden, 1998; Muchimapura et al., 2002; Schiller et al., 2003; Schiller et al., 2006; Bibancos et al., 2007; Ago et al., 2013; Dankoski et al., 2014). In agreement with this literature, densities of active serotonergic neurons in the current study were lower in mice housed in postweaning isolation (Figure 2C). This effect was only observed within the DRD, the subdivision in the dorsal midline region of the DRN. Although lower densities of serotonergic neurons in the lateral subregions of the DRN (the DRL and PDR) could decrease the power to detect the effects of social isolation, no effect of social isolation was observed in the DRV, a ventral midline region with a high density of serotonergic neurons. The decreased activity in isolated individuals is similar to a finding of decreased serotonergic activity in the DRN following a resident-intruder social interaction in socially isolated adult mice (Lv et al., 2022). Overall, this difference among groups suggests that (1) the DRD is particularly sensitive to the manipulation of social isolation and (2) DRD neurons decrease their responsiveness to opposite-sex social interaction following isolation in both males and females.

In contrast to this clear decrease in c-Fos expression by serotonergic neurons, postweaning isolation had mixed effects on vocal and nonvocal behaviors during the social interaction. Subtypes of USVs, a type of call made mostly by male mice in opposite-sex interactions (Neunuebel et al., 2015; Sterling et al., 2023), and which attract females (e.g., Hammerschmidt et al., 2009; Asaba et al., 2014), showed different effects of isolation. Harmonic calls, which are elevated in number around male mounting (Hanson and Hurley, 2012), were significantly increased only in the isolated male group, while there was no difference in the number of non-harmonic calls across treatment groups. This selectivity reflects a similar targeted effect of postweaning isolation on harmonic calls during male–male interactions (Keesom et al., 2017a), and are similar in general to previous reports that either adult or juvenile isolation may alter vocalization number and structure, in conjunction with effects on a range of neurochemical systems (Tomazini et al., 2006; Bassi et al., 2007; Chabout et al., 2012; Keesom et al., 2017a; Hamed and Kursa, 2020; Lefebvre et al., 2020; Zhao et al., 2021; Caruso et al., 2022; Broadfoot et al., 2023). As expected for a signal related to sexual behavior, the increase in harmonic vocalizing in the current study was accompanied by elevated mounting in the isolated male group. These findings suggest the interesting possibility that social isolation differentially targets vocal regulatory circuits for harmonic versus nonharmonic calls.

In parallel with the elevation of harmonic calls and mounting, the production of BBVs, often used by females in conjunction with defensive kicking (Wang et al., 2008; Sugimoto et al., 2011; Finton et al., 2017), were also higher in the isolated male group than the other groups. This is similar to a finding in male–male interactions (Keesom et al., 2017b). Although numbers of kicks by females were highest in number in both the isolated male group and the isolated female group, this difference was not significant. These findings together suggest that social isolation of males causes increased social/sexual pursuit of females, with females responding with greater levels of defensiveness. A behavior that does not necessarily fit this narrative is digging behavior, which was decreased for both isolated males and isolated females. Digging has been described as an anxiety-like or perseverative behavior (Thomas et al., 2009), so this finding may be consistent with the increased anxiety-like behavior observed in rodents following social isolation (Huang et al., 2017).

### Effects of social isolation within groups

Behavioral correlations with c-Fos expression by serotonergic neurons reveal additional similarities and differences among social treatment groups and between sexes. There was a striking inverse correlation between the density of double labeled neurons in the DRD and the proportion of USVs that were in the harmonic versus nonharmonic categories, but only for the isolated male group. Since males are the predominant producers of USVs during opposite-sex interactions (Neunuebel et al., 2015; Sterling et al., 2023), this finding suggests that c-Fos expression by serotonergic neurons is inversely associated with harmonic calling for isolated males but not socially housed males. A caveat to this conclusion is that the isolated male group showed a much wider variation in the harmonic ratio than other social groups. Harmonics are usually a minority of USVs produced (Hanson and Hurley, 2012; Keesom et al., 2017a; Hood et al., 2023), but some isolated males produced even more harmonic than nonharmonic calls, and some isolated males produced no harmonic calls at all. This wide variation may therefore have provided a substrate for observing a correlation that did not exist for the other social treatment groups. Similar to our finding, social interactions in rats trigger release of a range of neurochemicals including serotonin in different brain regions, in correlation with the production of USVs, which are higher in previously isolated individuals (Hamed and Kursa, 2020). In another study, pro-social 50 kHz calls of rats were positively correlated with the total tissue content of serotonin in several brain regions including amygdala, hippocampus, and medial prefrontal cortex (Hamed and Kursa, 2018).

Nonvocal behaviors during the social interactions were also correlated with the activity of serotonergic neurons in the DRD. The second dimension of a PCA, which showed strong positive loading of female kicking and male rearing and strong negative loading of male mounting and female anogenital investigation, correlated with c-Fos expression in the DRD in ways that varied across social treatment and sex. Both male subject groups showed correlations between the density of double labeled neurons and Dimension 2, but in different directions, with a positive correlation for isolated males and a negative one for socially housed males. Further, there was a sex difference, with isolated females and isolated males showing correlations with Dimension 2 that were the reverse of each other.

Previous studies have also reported correlations in social behavior with different measures of serotonergic activity. A previous study on c-Fos activity in the DRD of socially housed females found a positive correlation with male mounting and anogenital investigation, complementing the negative correlation with females kicking males seen in the current study (Petersen et al., 2020). Increases in calcium in DRN neurons is likewise related in time to mounting behavior in male mice paired with females (Li et al., 2016). Although the expression of c-Fos can capture behaviorally meaningful activity in neuron populations (Lyons and West, 2011; Pettit et al., 2022), the measurement of c-Fos in DRD neurons does not necessarily have a linear relationship to serotonin release in target tissues. One reason for this is that we measured numbers of serotonergic neurons that expressed threshold levels of c-Fos protein, a measurement that could mask variation in the levels of c-Fos expressed across serotonergic neurons. Second, although c-Fos is responsive to neural depolarization, c-Fos expression may be more sensitive to changes in inputs than to constitutive activity, so may not equally represent all firing modes of serotonergic neurons (Kovács, 2008). Lastly, c-Fos expression is not temporally precise, so that it may only imperfectly reflect transient neural events (Kovács, 2008). Nevertheless, our current results are consistent with an earlier study that directly measured serotonin within a target of the DRD, the inferior colliculus (Klepper and Herbert, 1991; Keesom and Hurley, 2016; Niederkofler et al., 2016; Petersen et al., 2020). In this study, serotonin in the IC of males correlated inversely with female BBVs associated with kicking and lunging at males, matching the trend for socially housed males in the current study. All of these results across studies in general confirm that the serotonergic system, and specifically neurons within the DRD, are sensitive to the valence of opposite-sex interaction, and that these relationships vary depending on social history and between sexes. These relationships, repeated across multiple studies, further suggest that the behavioral and neural changes we observed are related to each other in some way.

### Regulation of social plasticity

For social animals, behavioral flexibility is illustrated in the display of different communicative behaviors across social contexts (Ota et al., 2018; Zala et al., 2020), but even within specific contexts, the interaction of social participants with different life histories and internal states can create a wide range of variation in social outcomes. Both between-context and within-context variation in behavior are accompanied by covariation in neural activity in brain regions that respond to social signals or regulate social behavior (Vignal et al., 2005; Kelly and Goodson, 2014; Field and Maruska, 2017; Forlano et al., 2017; Alger et al., 2020; Anderson et al., 2023), For example, activation of neurons expressing vasotocin, mesotocin, or catecholamines in nuclei in the social behavior network of zebra finches correlates with social behavior, anxiety-like behavior, and dominance and partner-directed behavior in ways that differ with the sex of the focal individual, and with the familiarity and sex of the social partner (Kelly and Goodson, 2014, 2015). Expression of tyrosine hydroxylase in starlings in the nucleus accumbens and ventral tegmental area correlate with vocal behaviors and the motivation to join a group (Maksimoski et al., 2023). In midshipman fish, correlations among nuclei in the social decision making network are greater in response to acoustic courtship signals than agonistic grunts (Ghahramani et al., 2018). Social isolation has been viewed as reducing these types of social plasticity, in that individuals show behaviors like aggression or persistent investigation or physical contact that are not graded depending on the social context or partner cues (Tóth et al., 2008; Keesom et al., 2017a).

Some of our findings are consistent with a decreased role for the serotonergic system in context-dependent regulation of neural target regions following social isolation. In addition to fewer serotonergic neurons being activated during social interactions in previously isolated versus socially housed animals (Lv et al., 2022, current study), other measures of serotonergic function are also decreased following isolation. In the inferior colliculus, a midbrain auditory nucleus that is a target of serotonergic DRD neurons, serotonergic axon density is lowered following juvenile social isolation (Klepper and Herbert, 1991; Niederkofler et al., 2016; Keesom et al., 2018; Petersen et al., 2020). This difference is only seen in females; males have consistently lower axon densities regardless of postweaning social experience (Keesom et al., 2018). Directly measured serotonergic increases during same-sex interaction also take longer to peak for socially isolated males than for socially housed males, and across-individual correlations between serotonin levels and social behavior that exist in the socially housed group are not present in the isolated group (Keesom et al., 2017b). Likewise, serotonergic increases measured with a chemogenetic sensor in the prefrontal cortex are lower in previously isolated than previously socially housed males (Lv et al., 2022). In contrast to these decreases in serotonergic activity, c-Fos activation in the IC in response to pharmacologically elevated serotonin actually increases following isolation, potentially due to changes in receptor expression (Davis et al., 2021). Although this effect seems to represent greater serotonergic regulation of the target neurons, it could represent a compensatory response to upstream decreases in serotonergic signaling.

Behaviorally, the manipulation of serotonin has effects on vocal signals that parallel the effects of social isolation. Systemic injection of a serotonin precursor in socially housed male mice significantly decreases the proportion of harmonic vocalizations but not overall vocalization number, suggesting a focused effect of serotonin on harmonic vocalizations (Hood and Hurley, 2024). This finding is consistent with the correlation between decreased c-Fos expression and increased harmonic USV production in the isolated males in the current study. Increased serotonin levels furthermore cause males to be more responsive to female signals, in that they suppress USV production more in response to BBV playback when serotonin levels are elevated (Hood and Hurley, 2024). Implicating isolation in responses to BBVs, males that have been isolated in adulthood recover from USV suppression more quickly than socially housed males, although whether serotonin is implicated in this difference has not been directly tested (Hood et al., 2023). Taken together, this body of work suggests a reduced sensitivity of the serotonergic system to social cues following isolation, which may alter the responses of males to female social cues.

Unlike the correspondence between serotonin and behavior across groups, the correlations between c-Fos expression and behavior within groups do not neatly fit a narrative of reduced serotonergic regulation of communication following social isolation. This is because the density of active DRD neurons correlates with behavior in both socially housed and isolated males, albeit in opposite directions. In females, there was even a correlation between serotonergic activation and behavior in the isolated but not the social group. The broad scale of inputs to the DRN (Pollak Dorocic et al., 2014; Rood and Beck, 2014; Weissbourd et al., 2014), as well as the widespread projections from the DRN to sensory, motor, and integrative brain regions (Vertes, 1991; Lee et al., 2008; Muzerelle et al., 2016; Niederkofler et al., 2016; Steinbusch et al., 2021) make the functional consequences of these correlations difficult to interpret. For example, AVP receptor-expressing DRN neurons, some of which are serotonergic, are sensitive to the valence of social interactions, so it is possible that isolation-induced changes in the correlations between c-Fos expression by serotonergic neurons and behavior represent an altered valence of social cues (Patel et al., 2022). DRN neurons are not only sensitive to social cues, however, but also influence social behaviors, including aggressive and sexual behavior (Kiser et al., 2012; Takahashi and Miczek, 2013; Rubio-Casillas et al., 2015; Niederkofler et al., 2016). Isolation-induced changes in correlations between c-Fos expression and behavior could therefore also represent a changing balance of the causal connections among social cues, serotonergic activity, and social behavior. Disentangling these functional pathways could be advanced through future experiments in which social cues are carefully controlled.

On a broader scale, our findings support the concept of the “tuning” of neuromodulatory and neurochemical systems by instructive life experiences. Social animals like mice may live at a wide range of population densities, from a few individuals invading a new habitat to extreme population explosions (Nathan et al., 2015; Joosse, 2021). For species like this, early life social isolation could be instructive on the social conditions that individuals are likely to encounter in adulthood. In another species that undergoes cycles in population density, prairie voles (Blondel et al., 2016), juvenile social isolation and the stressor of chronic social defeat do not alter the level of expression of nonapeptide and opioid receptors in the lateral septum, but increase the correlations in expression level, potentially allowing greater coordination in activity among these regions (Sailer et al., 2022). Other kinds of behaviorally relevant experiences can also alter neurochemical regulation. For juvenile female zebra finches, the experience of hearing adult song alters the sensitivity of dopaminergic neurons in the ventral tegmental area to courtship versus non-courtship song (Barr and Woolley, 2018). In communally breeding spiny mice, the presence or absence of non-related individuals in early life alters the responses of lateral septal neurons toward conspecifics (Wallace et al., 2023). In prairie vole females raising pups, the experience of a present versus absent male parent alters the correlation between the numbers of neurons expressing vasopressin in the supraoptic and paraventricular nuclei of the hypothalamus haand pup grooming (Hiura et al., 2023). These experiences all relate to salient aspects of species-specific social structure. However, understanding whether these types of plasticity are adaptive or represent pathological responses to the lack of a normal social environment will require assessing the fitness consequences of differing behavioral phenotypes across social environments.

## Data Availability

The original contributions presented in the study are included in the article/Supplementary material, further inquiries can be directed to the corresponding author.

## Supplementary material

Supplementary Figure S1 **(A)** In the DRD and DRV, there are significantly more TPH+ neurons in isolated males than isolated females. **(B)** This is reflected in the higher numbers of TPH+ neurons in isolated males than socially housed males in the DRD and DRV.

Supplementary Figure S2 Percentages of neurons labeled for antibodies to both c-Fos and TPH relative to those labeling for c-Fos only in four subregions of the dorsal raphe nucleus. The four subject groups of individually versus socially housed females and males are represented for each subregion. DRD, dorsomedial dorsal raphe; DRV, dorsoventral dorsal raphe; DRL, dorsolateral dorsal raphe; PDR, posterodorsal raphe.

Supplementary Table S1 Spearman correlations between calls and non-vocal behaviors. Red text indicates significant correlations after correction for multiple comparisons. “m” designates male behaviors and “f” designates female behaviors. “iag” indicates anogenital investigation and “inn” indicates nose-to-nose investigation. “harm” = harmonic USV, “nonharm” = nonharmonic USV; “BBV” = broadband vocalization, or squeak.
