# Supplementary figures and images for: Solitude and serotonin: juvenile isolation alters the covariation between social behavior and cFos expression by serotonergic neurons
Source: Front Neurosci. 2024 Oct 22;18:1446866. doi: 10.3389/fnins.2024.1446866 (PMC11535725; doi:10.3389/fnins.2024.1446866)

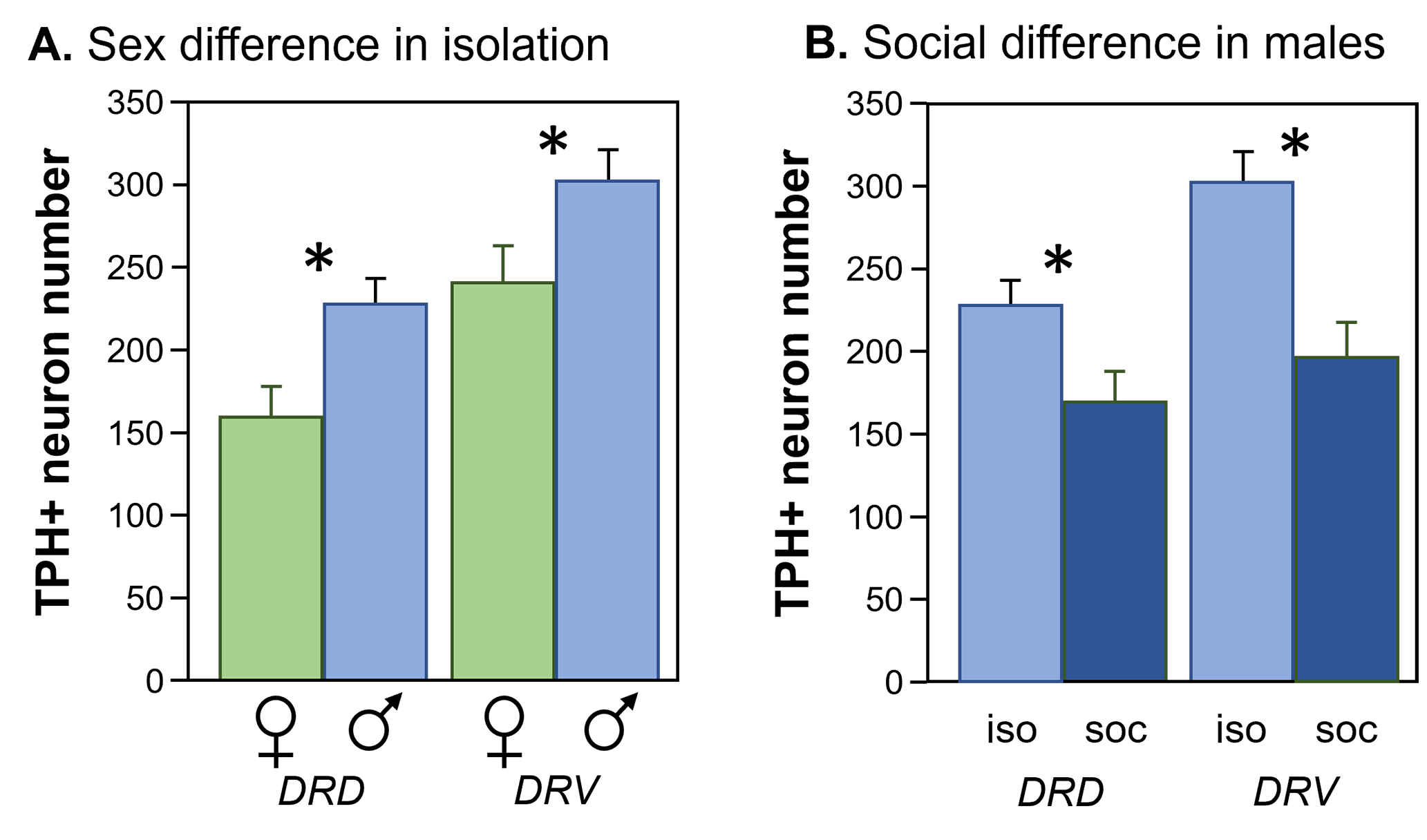

Supplement: Supplementary Figure S1 — (A) In the DRD and DRV, there are significantly more TPH+ neurons in isolated males than isolated females. (B) This is reflected in the higher numbers of TPH+ neurons in isolated males than socially housed males in the DRD and DRV. [file Image_1.TIF]

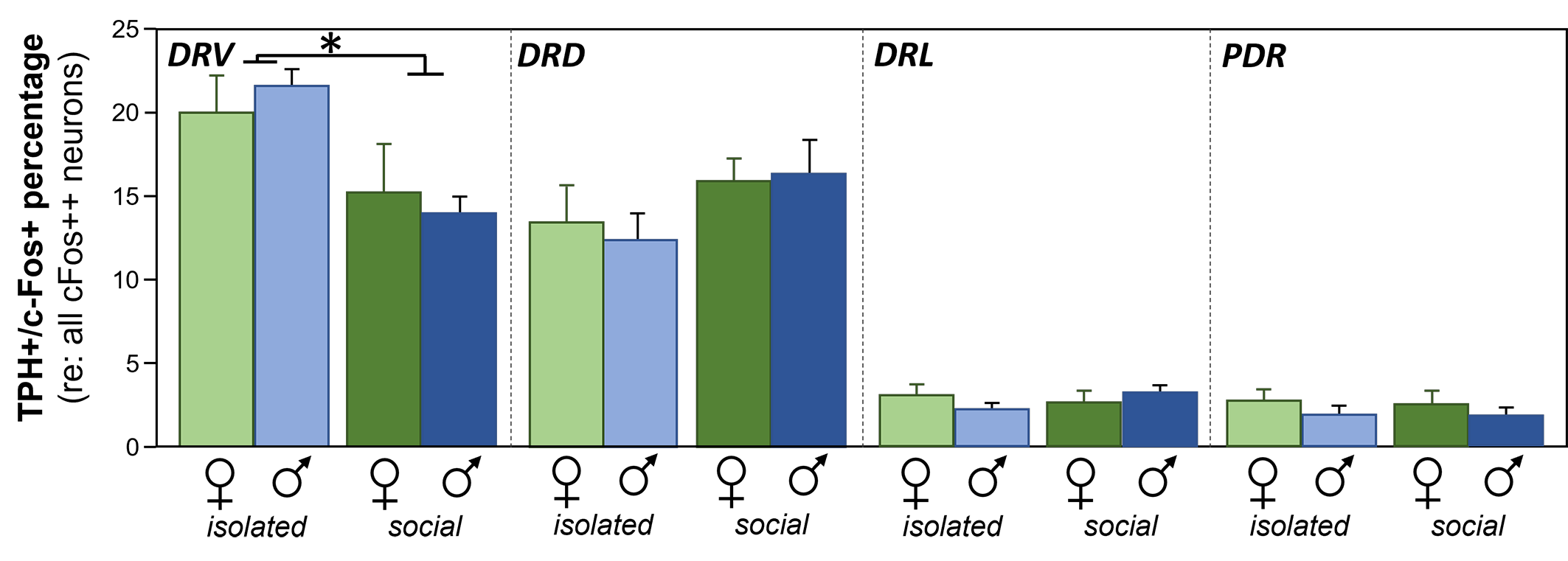

Supplement: Supplementary Figure S2 — Percentages of neurons labeled for antibodies to both c-Fos and TPH relative to those labeling for c-Fos only in four subregions of the dorsal raphe nucleus. The four subject groups of individually versus socially housed females and males are represented for each subregion. DRD, dorsomedial dorsal raphe; DRV, dorsoventral dorsal raphe; DRL, dorsolateral dorsal raphe; PDR, posterodorsal raphe. [file Image_2.TIF]
